# Increased functional connectivity patterns in mild Alzheimer’s disease: A rsfMRI study

**DOI:** 10.3389/fnagi.2022.1037347

**Published:** 2023-01-09

**Authors:** Lucía Penalba-Sánchez, Patrícia Oliveira-Silva, Alexander Luke Sumich, Ignacio Cifre

**Affiliations:** ^1^Facultat de Psicologia, Ciències de l’educació i de l’Esport, Blanquerna, Universitat Ramon Llull, Barcelona, Spain; ^2^Human Neurobehavioral Laboratory (HNL), Research Centre for Human Development (CEDH), Faculdade de Educação e Psicologia, Universidade Católica Portuguesa, Porto, Portugal; ^3^NTU Psychology, School of Social Sciences, Nottingham Trent University, Nottingham, United Kingdom

**Keywords:** Alzheimer’s disease, mild cognitive impairment, functional connectivity, dynamic functional connectivity, point process analysis, resting state fMRI

## Abstract

**Background:**

Alzheimer’s disease (AD) is the most common age-related neurodegenerative disorder. In view of our rapidly aging population, there is an urgent need to identify Alzheimer’s disease (AD) at an early stage. A potential way to do so is by assessing the functional connectivity (FC), i.e., the statistical dependency between two or more brain regions, through novel analysis techniques.

**Methods:**

In the present study, we assessed the static and dynamic FC using different approaches. A resting state (rs)fMRI dataset from the Alzheimer’s disease neuroimaging initiative (ADNI) was used (*n* = 128). The blood-oxygen-level-dependent (BOLD) signals from 116 regions of 4 groups of participants, i.e., healthy controls (HC; *n* = 35), early mild cognitive impairment (EMCI; *n* = 29), late mild cognitive impairment (LMCI; *n* = 30), and Alzheimer’s disease (AD; *n* = 34) were extracted and analyzed. FC and dynamic FC were extracted using Pearson’s correlation, sliding-windows correlation analysis (SWA), and the point process analysis (PPA). Additionally, graph theory measures to explore network segregation and integration were computed.

**Results:**

Our results showed a longer characteristic path length and a decreased degree of EMCI in comparison to the other groups. Additionally, an increased FC in several regions in LMCI and AD in contrast to HC and EMCI was detected. These results suggest a maladaptive short-term mechanism to maintain cognition.

**Conclusion:**

The increased pattern of FC in several regions in LMCI and AD is observable in all the analyses; however, the PPA enabled us to reduce the computational demands and offered new specific dynamic FC findings.

## Introduction

Alzheimer’s disease (AD) is the most prevalent progressive neurodegenerative disease associated with age. It typically starts with a preclinical stage and progresses through mild cognitive impairment (MCI) to clinically relevant AD (i.e., dementia-type AD). Although great efforts have been made to identify AD biomarkers, AD remains a clinical diagnosis. Early and accurate prediction of the disease remains limited. To address the increasing burden of AD, the dynamic brain changes associated with shifts in cognitive function that underpin what causes dementia must be understood. Abnormal brain connectivity has been observed 20 years prior to the onset of brain atrophy and clinically relevant symptoms of AD (Ashraf et al., 2015; Nakamura et al., 2017). Thus, the relative risk of the development of MCI and dementia might be determined based on resting state functional connectivity (FC). It is, therefore, critical to thoroughly understand aberrant FC at each stage of the disease in order to improve strategies for early intervention.

Resting-state functional magnetic resonance imaging (rsfMRI) data, acquired while the participants are awake without performing any task, can be used to assess intrinsic brain functional connectivity. By means of this high spatial resolution neuroimaging technique, the blood-oxygen-level-dependent (BOLD) signal across brain regions is quantified (Yamasaki et al., 2012; Sporns, 2013; Liu et al., 2015; Xue et al., 2019). Some investigators have analyzed the BOLD signals using graph analytical methods to explore the network’s topological features of patients with AD (Toussaint et al., 2014; Jie et al., 2016; Bhuvaneshwari and Kavitha, 2017; Xu et al., 2020). For instance, the characteristic path length supposedly reflects the functional integration of brain networks. Hence, a shorter path length indicates efficient communication between regions. On the other hand, the clustering or modular coefficients provide information regarding the segregation of the networks, i.e., the degree of specialization of brain regions. Seminal research revealed a decreased path length and clustering coefficients in AD in comparison to healthy patients (Supekar et al., 2008; Krienen and Buckner, 2009). Other studies reported a decrease in clustering degree and modularity in AD (Brier et al., 2014) and MCI (Seo et al., 2013) but a similar characteristic path length relative to individuals without MCI or AD.

Functional connectivity and dynamic functional connectivity (dFC) are measures of signal synchronicity that allow researchers to analyze the gradual and continuous changes of the BOLD signal, which are represented by signal correlations of the whole signal or selected windows, respectively (for a detailed technical description refer to Chen et al., 2017; Keilholz et al., 2017; Scarapicchia et al., 2018; Zhang et al., 2020). Three metanalyses (Jacobs et al., 2013; Li et al., 2015; Badhwar et al., 2017) and another study (Kim et al., 2016) using FC revealed a decrease in default mode network (DMN) connectivity in AD, mostly involving the precuneus (PCu) and the posterior cingulate cortex (PCC). These areas are implicated in episodic memory and attentional processing and are typically affected in AD (Jacobs et al., 2013). In MCI, the results are less consistent. Some studies have found an increase in FC in the mentioned regions, while others have found the opposite. Additionally, increased limbic connectivity has been seen in MCI (Badhwar et al., 2017), and increased connectivity of the salience network (SAL) has been observed in both MCI and AD (Wang et al., 2013; Thomas et al., 2014). Such inconsistent findings in FC may reflect the heterogeneity of MCI subtypes, which might be differentiated, for example, by symptoms and the extent of illness progression (Badhwar et al., 2017).

Additionally, seminal researchers have been discussing that an increase in functional connectivity between brain regions in MCI and early stages of AD has been seen to take place when communication between specific brain regions is impaired. This has been interpreted as reflecting the recruitment of alternative paths within the system (Hillary and Grafman, 2017; Marek and Dosenbach, 2018; Oldham and Fornito, 2019). The DMN, SAL, and frontoparietal network (FPN) are networks that have been reported to become hyperconnected at some stage during disease progression. These multimodal networks connect several regions and integrate information processing, providing high value at a high cost (Hillary and Grafman, 2017; Marek and Dosenbach, 2018). An increase in FC between alternative paths is efficient and adaptive in short term. However, rich hubs are a perfect place for beta-amyloid deposition, which can lead to secondary damage caused by metabolic stress and the eventual breakdown of the system (Hillary and Grafman, 2017). Thus, hyperconnectivity that takes place at the beginning of many neurodegenerative diseases may be followed by hypoconnectivity between these recruited paths and cognitive decline as the illness progresses (Marek and Dosenbach, 2018).

A further source of inconsistent FC findings in MCI and AD may be the use of different methods and parameters. For instance, the preprocessing steps, parcellation methods, window size used, *p*-values, number of samples, or inclusion and exclusion criteria (Tam et al., 2015; Badhwar et al., 2017). With regards to samples, studies that used exploration methods (e.g., correlation, clustering algorithms, or matrix decomposition) and other statistical methods (e.g., *t*-test, ANOVA, and Bayesian inference) typically do not compare across the finer stages of illness (e.g., HC, EMCI, LMCI, and AD), although some machine learning studies have done so. There have, however, been comparisons of HC, MCI, and AD (Zhang et al., 2020); HC, EMCI, and LMCI (Cai et al., 2015; Lee et al., 2016); HC with AD (Zheng et al., 2017); HC with EMCI (Zamani et al., 2021); HC with amnestic MCI (Wang et al., 2013; Jie et al., 2016); and HC with amnestic vs. non-amnestic MCI. Nevertheless, the precise trajectory for FC from HC to AD remains unclear.

One of the limitations of traditional FC approaches is the high demand for computational processing and sensitivity to residual noise. As the BOLD signal usually presents the same stereotypical pattern, larger amplitude BOLD signal peaks likely provide the most critical information, i.e., neural events (Aguirre et al., 1998; Cifre et al., 2020). Several studies suggest that patients with neurological and psychiatric diseases present greater variability in the BOLD signal over the scan session (Keilholz et al., 2017). Hence, these peaks might be highly useful in these cases as they might reveal key intrinsic brain connectivity that can only be detected when assessing the amplitude of the signals (Keilholz et al., 2017). In fact, a higher recognition accuracy between healthy participants and patients with autism has already been detected in our previous work (Cifre et al., 2021) using the point process analysis (PPA), a method that captures these relevant events. Other researchers obtained similar results, thereby successfully differentiating across groups when applying the PPA to patients with diabetes (Li et al., 2014).

Using the PPA method, local peaks of the BOLD time series are selected to generate a co-activation matrix that defines the co-occurrence of points. Apart from reducing noise, the bursts of correlated activity between regions are not required when using the PPA. This phenomenon occurs because the assumption behind this method is that those events with a higher amplitude, i.e., peaks, contain avalanches of neural information that are the consequence of the intrinsic activity between communities of neurons (Cifre et al., 2021). Previous work exploring the BOLD activation showed similar results when using a seed-based approach and a PPA, i.e., activation maps when using a seed-based approach, selecting all the time points (between 140 and 240), in comparison to using only a PPA, selecting just those time points that surpass 1 SD of the BOLD signal (between 4 and 8 points). Additionally, when exploring the changes in brain integration or connectivity, the PPA is more sensitive to capturing changes across groups (Tagliazucchi et al., 2011 2012; Hutchison et al., 2013; Cifre et al., 2020 2021). To the best of our knowledge, PPA has never been applied to rsfMRI datasets of patients with AD and MCI. This method might offer an efficient way to manage big data sets and to better understand the changes in the FC dynamics across the different stages of the disease.

The main goal of this present study was to explore FC and dFC across groups. A PPA was applied to a dataset of patients with AD, LMCI, EMCI, and age- and sex-matched healthy individuals (HC). Additionally, to compare findings with other classical methods, pairwise correlations of the whole BOLD signal, graph theory measures, and an SWA were applied. In line with previous literature, it was expected to find (1) differences across the four groups in FC and variability within and between the DMN, SAL, visual networks (*VS*), and CEN; (2) an increased FC in EMCI and LMCI and a slightly decreased FC in AD in several networks in contrast to the other groups; and (3) more subtle differences across groups when using the PPA in comparison to the other methods.

## Materials and methods

### Participants

All rsfMRI, T1 MRI, and demographic data from participants were downloaded from the Alzheimer’s Disease Neuroimaging Initiative (ADNI; http://adni.loni.usc.edu/; Franzmeier et al., 2017). A total of 36 HC age- and sex-matched, 29 with EMCI, 30 with LMCI, and 34 with AD were included in this study. All the images correspond to the screening visit, which is coded in ADNI2 as Screening MRI-New Pt (V02). Between groups, there were no differences in age, sex, and years of education. Patients with AD presented significantly lower scores in the screening assessment cognitive test Mini-Mental State Examination (MMSE) in comparison with the three other groups. Expectedly, there were no differences between groups in episodic memory measured by the Scale Logical Memory II (delayed paragraph A recall) and the Wechsler Memory Scale cognitive tests (refer to Table 1). A participant from the HC group was eliminated before the analysis because some time series were missing. Further inclusion and exclusion criteria are exposed in detail in the “ADNI 2 Procedures manual” (pages 27–30); access is available through this link: http://adni.loni.usc.edu/wp-content/uploads/2008/07/adni2-procedures-manual.pdf. Data access was approved by contacting the ADNI Ethics Committee^1^ and sending a request with a proposed analysis and the name of the principal investigators.

**Table 1:** Information of the participants included in this study.

	Group	Between-group differences				
	HC	EMCI	LMCI	AD	*f*	Value of *p*
Number	35	29	30	34	–	–
Sex (M/F)	(15/21)	(13/16)	(18/12)	(16/18)	–	Chi^2^ = 2.21 *p* = 0.52
Age $\bar{x}$ (SD)	73.60 (5.75)	71.36 (5.46)	70.63 (8.23)	72.44 (7.09)	1.08	*p* = 0.36
Years of ed. $\bar{x}$ (SD)	16.37 (2.36)	15.96 (2.41)	16.85 (2.60)	15.5 (2.75)	1.72	*p* = 0.167
MMSE $\bar{x}$ (SD)	28.53 (1.92)	27.76 (1.94)	27.60 (1.47)	22.69 (2.53)	54.5	*p* < 0.0001^a^^,^^b^^,^^c^
LDELT $\bar{x}$ (SD)	13.50 (3.48)	8.65 (1.80)	4.46 (2.92)	1.31 (1.97)	122.28	*p* < 0.0001^a^^,^^b^^,^^c^^,^^d^^,^^e^^,^^f^
MOCA $\bar{x}$ (SD)	25.49 (1.91)	24.11 (2.50)	21.61 (3.69)	16.5 (5.24)	38.45	*p* < 0.0001^a^^,^^b^^,^^c^^,^^d^^,^^f^

The table displays means, standard deviations and differences in age, years of education (Years of ed.), scores in the Mini Mental State Examination (MMSE), in the Scale Logical Memory II (delayed paragraph A recall) (LDELT) and in the Montreal cognitive assessment (MOCA). Statistical analysis of variance (ANOVA) was used to assess differences. Differences in gender were assessed using a Chi Squared test. HC, healthy controls; EMCI, early mild cognitive impairment; LMCI, late mild cognitive impairment; AD, Alzheimer’s disease.

a AD < EMCI.

b AD < LMCI.

c AD < HC.

d EMCI < LMCI.

e EMCI < HC.

f LMCI < HC.

### Data acquisition

T1 and fMRI images were acquired using Philips Medical Systems Scanners and they underwent control at the Mayo Clinic. The fMRI images were obtained with a field strength of 3.0 Tesla, a repetition time of 3 s, an echo time of 30 ms, a flip angle of 80°, matrix 64 × 64, 140 volumes, 48 slices per volume, and a slice thickness of 3.3 mm. The voxel size was 3.3 × 3.3 × 3.3 mm^3^. For further details on MRI acquisitions, refer to the “MRI scanner protocol” at http://adni.loni.usc.edu/wp-content/uploads/2010/05/ADNI2_MRI_Training_Manual_FINAL.pdf.

### Data preprocessing

The MATLAB toolbox “Data Processing Assistant for Resting-State fMRI” (DPARSF; Yan and Zang, 2010) was used to preprocess the data. First, slice timing and head motion correction were applied. No subjects with a mean movement on translation or rotation over 2 mm were found. Then, registration was performed for the corrected fMRI image. Finally, normalization using the Montreal Neurological Institute (MNI) space, spatial smoothing (with an 8 mm full-width half-maximum Gaussian Kernel), and bandpass filtering (0.001–0.1 Hz) to remove low-frequency scanner drift and physiological noise of the fMRI images were applied.

The automated anatomical labeling (AAL) atlas (Tzourio-Mazoyer et al., 2002) was used to extract 116 Regions-Of-Interest (ROIs) of the preprocessed rsfMRI dataset. This parcellation method has been shown to be optimal to understand the FC between brain regions (Arslan et al., 2018). The voxels within each ROI were averaged to obtain a time series per ROI. Each time series contains 140 timepoints (3 s TR, i.e., 420 s in total).

**Figure 1:**
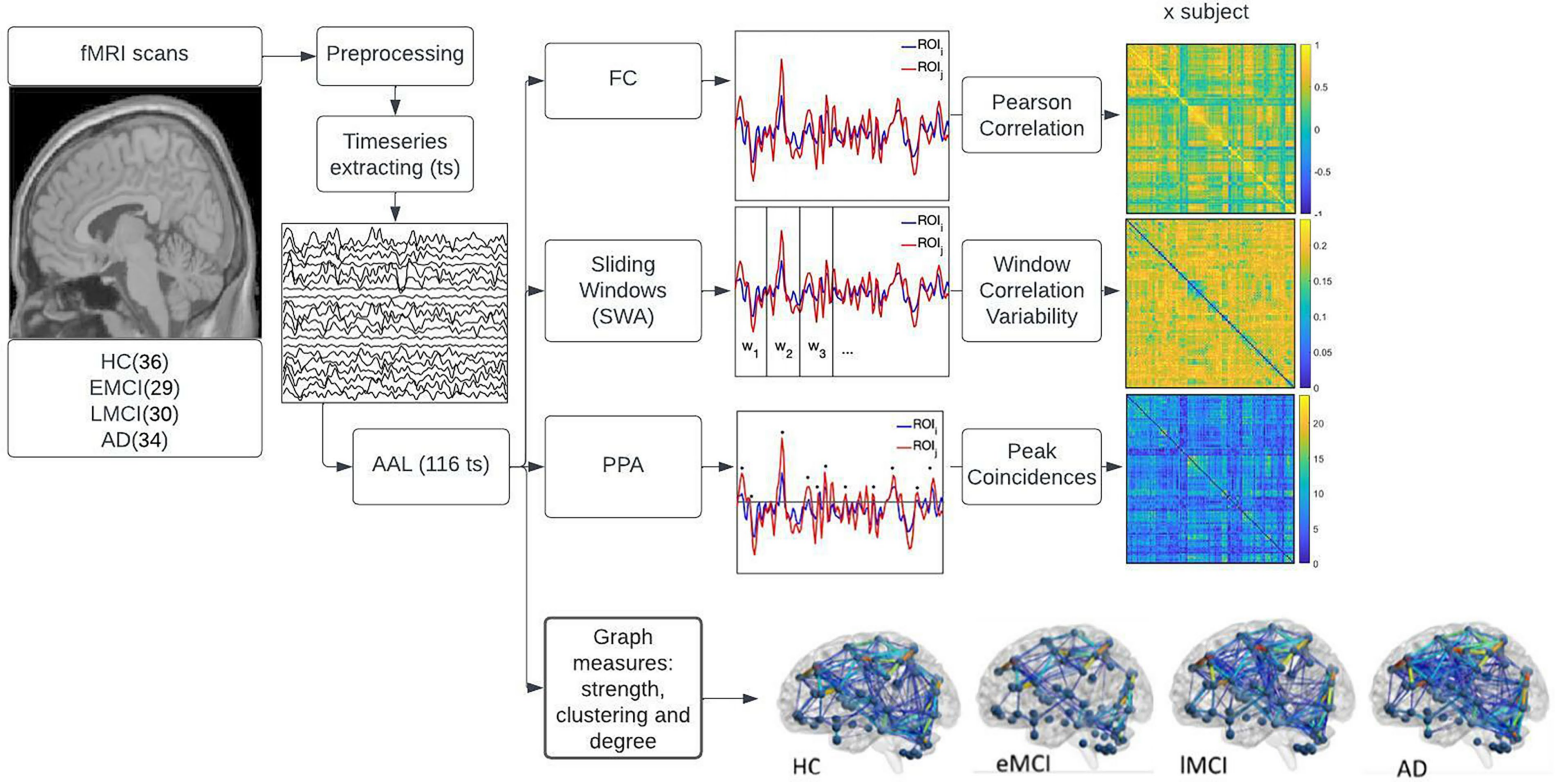
displays the methods used in the study. First, the fMRI and T1 image acquisitions were downloaded from the Alzheimer’s disease neuroimaging initiative (ADNI) database. Then the images were preprocessed using the DPARSF pipelines. Timeseries from 116 regions of interest (ROIs) were extracted using the automated anatomical labeling (AAL) atlas. These 116 time series were used to perform three main analyses displayed on the right side of the figure namely, functional connectivity (FC), sliding window analysis (SWA), and point process analysis (PPA). The top right side of the figure shows the FC where the Pearson’s correlation coefficient of each pair of regions was computed using the mean whole signal of each time series; the middle right side of the figure shows the SWA, that consisted of dividing the time series into non-overlapping windows, computing the FC for each window and determining the variability in FC across windows; the third plot on the right side of the image shows the PPA, this is a single frame analysis where points that surpass the threshold of 1 SD of each time series were selected, coincident points between pair of regions where summed and displayed in a matrix of addition. The fourth plot on the right side of the image displays the graph measures conducted in the study. To test the statistical significance of each analysis, a one-way-ANOVA with multiple comparison tests was conducted, corrected with Bonferroni at *p* < 0.05.

All the analyses described in the following subsections (refer to Figure 1) were performed using MATLAB ver. R2018a.

### Static FC analysis with Pearson’s correlation

The Pearson’s correlation coefficient (PCC) between the entire time series was computed to extract the FC in a pairwise manner (ROI to ROI correlation) per participant. Hence, correlation matrices of 116 × 116 (ROI * ROI) were obtained per each subject. Then, a one-way analysis of variance (ANOVA) and *post-hoc* comparisons were conducted to explore differences between the groups. Those regions that presented differences in FC across groups were checked for outliers; more specifically, whether the FC was three-scaled median absolute deviations (MAD) away from the median for each participant in each group was explored. Then the FC was computed again after removing these participants.

### Graph theory measures

To explore some characteristics of the brain networks, such as integration and segregation, several graph theory measures were computed (Rubinov and Sporns, 2010). More specifically, we computed the clustering coefficient to measure local connectivity and segregation, the global path length to measure the average shortest path between two nodes and to reveal how efficient communication between regions or how integrated the brain is, and finally, the mean degree to quantify the mean number of edges connected to a node, i.e., mean of all node degrees. These measures were computed by converting the FC matrices into binary graph matrices that represent nodes and edges.

The brain is a highly centralized network, often called a scale-free or a power-law network (Sporns et al., 2004; Haimovici et al., 2013). This type of structure shows an exponential or power relationship between the degree of connectivity of a node and its frequency of occurrence; more specifically, the brain contains a few rich hubs or nodes that connect to several regions, and many nodes are connected to just a few regions, i.e., the majority of nodes present a low degree. Taking this fact into consideration, to satisfy small worldness or scale freeness of features, different thresholds were used to binarize the static FC matrices before performing graph theory measures, i.e., raw FC values (PCC), from 0.1 to 0.5 at intervals of 0.01. A threshold of 0.3 was selected. Hence, those ROI-to-ROI connections that exceeded a threshold of 0.3 (PCC value) were set to 1, while those below 0.3 were set to 0 (note that the possible FC values range was −1 to 1, absolute values were not used). This threshold, apart from ensuring scale freeness, enables one to better differentiate among groups (refer to Supplementary material). Other researchers using rsfMRI datasets concluded that a threshold between 0.21 and 0.4 is optimal to enable the differentiation of groups (Aurich et al., 2015; Ye et al., 2015; Ahmadi et al., 2021; Ng et al., 2021). Graph theory measures were performed using the brain connectivity toolbox: https://sites.google.com/site/bctnet/.

After extracting the brain network features, a one-way analysis of variance (ANOVA) and *post-hoc* comparisons were conducted to explore differences between pairs of groups.

### Temporal variability of FC by means of sliding window correlation and standard deviation

**Figure 2:**
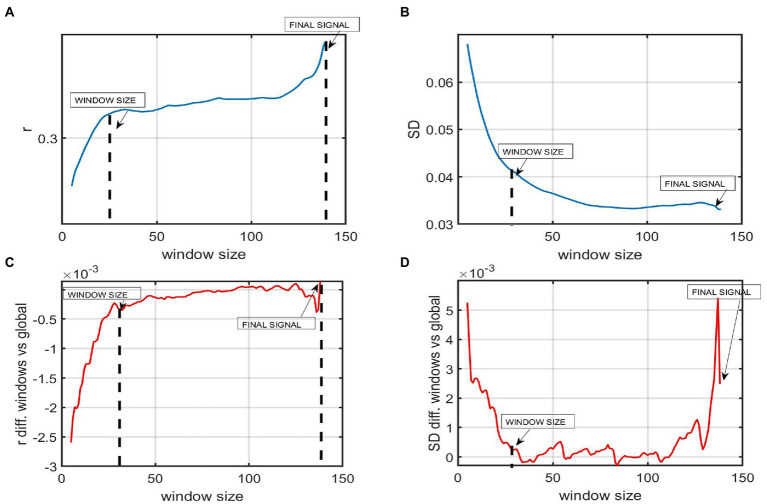
The figure represents the window size analysis before conducting the sliding window correlation analysis. **(A)** Global FC as a function of window size. The *Y*-axis represents the mean FC or correlation of all the ROI to ROI correlations windows of a participant with AD. The *X*-axis shows 140 time points of the whole time series. Each time point represents 3 s (TR = 3 s). The mean correlation varies as a function of window size. Shorter windows present a lower mean correlation while windows from 30 or above present a higher correlation. **(B)** The *Y*-axis represents the mean standard deviation (SD) of the SD of all the ROI to ROI correlations across windows of a participant with AD. The *X*-axis shows that this mean SD varies as a function of window size. Shorter windows present a higher variability while windows of 30 time points or above present a lower SD. **(C)** The plot shows the difference between the mean correlation of all ROI to ROIs using a certain window size and the global static **(D)** The difference in the variability among windows using window sizes higher than 30 fluctuates around 0, meaning that results in SWA are almost the same, no matter the window length chosen.

An SWA was used to explore the dFC of our samples, more specifically the temporal variability of FC. In order to use a window length that captured fast changes in the signal while also keeping an optimal level of robustness, a window size assessment was performed prior to the analysis (Leonardi and Van De Ville, 2015). This trade-off between sensitivity and specificity was examined by computing the mean correlation of the mean of all ROI-to-ROI correlations and exploring how the mean FC value varied as a function of window size (refer to Figure 2). The same procedure was used to explore the mean of the standard deviation (SD) of each window matrix as a function of window size. The length window assessment was performed for overlapping windows from 5 to 140 time points (i.e., from 15 to 420 s), with an increment of one time point (Mokhtari et al., 2019).

Once the optimal window size was selected, SWA was performed. First, the FC between each pair of regions was computed for each window, obtaining as many connectivity matrices as windows. Then, the variability (i.e., the standard deviation of FC across windows for each ROI-to-ROI) was computed, obtaining one covariance matrix per group.

A one-way analysis of variance (ANOVA) and *post-hoc* comparisons were conducted to explore differences between pairs of groups.

### Point process analysis

The main difference between static functional connectivity and PPA is that the former keeps all the time points for the analysis while the latter just includes those points that surpass a threshold of 1σ (Tagliazucchi et al., 2012; Cifre et al., 2020, 2021). The steps conducted in the PPA consisted of first thresholding the time series of each ROI considering the amplitude of the BOLD signal. Only those events or peaks that surpass 1σ were selected (refer to Tagliazucchi et al., 2012). Empirically, for the threshold of 1σ in a BOLD signal, we find on average 8.5 ± 2.8 upward crossings per 4 min of fMRI scan. This number of points has been proven to be sufficient by other authors (Tagliazucchi et al., 2012). This is because the BOLD signals that are situated upward are non-linear events that contain the most relevant information and follow a power law. Second, all those points of the time series that surpassed 1 SD of the BOLD signal were selected as relevant. Co-activation binary matrices for each timepoint were generated, e.g., in timepoint 1 FC between ROI 1 and ROI 2, the signal is relevant (both surpass 1 SD), in timepoint 1 ROI 1 and ROI 3 in time 1, the signal is not relevant (<1 SD…). This is conducted for all 140 time points for each of the 116 pairs or ROIs. Finally, a matrix of addition was computed for each pair of ROIs per participant, e.g., connectivity between ROI 1 and ROI 2 in PPA was acquired by adding all the time points that were relevant throughout the time series. This is similar to the correlation matrix obtained in the classic FC, but in the PPA, instead of computing the correlation between pairs of regions, the summation of the number of relevant points is obtained (refer to Supplementary material).

A one-way analysis of variance (ANOVA) and *post-hoc* comparisons were conducted to explore differences between pairs of groups.

### Statistical analysis

A one-way analysis of variance (one-way-ANOVA) and multiple comparison tests corrected with Bonferroni correction at *p* < 0.05 were used to compare results across groups in all the analyses conducted, i.e., FC, graph theory measures, SWA, and PPA. This method for multiple comparisons is one of the most commonly used for rsfMRI analysis (Lindquist and Mejia, 2015).

## Results

### Static functional connectivity

The results showed an increase in FC between several ROIs in LMCI and AD in comparison to HC and EMCI (refer to Table 2). More specifically, the AD presented an increased FC between the angular gyrus (left) and cerebellum crus II (left) in contrast to the EMCI and between the superior occipital gyrus (right) and calcarine fissure (left). Relative to HC, AD also presented an increase in FC between the thalamus and the inferior frontal gyrus (left), between the middle temporal gyrus (left) and the caudate nucleus (left), between the vermis 9 and the occipital superior (left), and between the caudate (right) and the frontal inferior gyrus (left). The only regions that presented a decreased FC in AD in comparison to HC were the middle temporal gyrus (right) and the superior occipital gyrus (left).

**Table 2:** Pairs of brain regions showing significant differences in FC between groups when performing a one-way ANOVA.

Network	ROI	Network	ROI	*P* value	*Post hoc* FC $\bar{x}$ (SD)
Cerebellum	Cerebellum: Crus II (left)	DMN	Parietal lobe	0.0007	AD > EMCI 0.38 (0.2) > 0.13(0.32)
	Cerebellum 2nd Non-motor: VIIB (right)	Sensory motor	Central region: Postcentral gyrus (left)	0.0021	AD > EMCI 0.47 (0.24) > 0.25 (0.23)
	Vermis 9	Visual II	Occipital superior (Left)	0.0022	AD > NC 0.34 (020) > 0.13 (0.25)
Visual I	Occipital lobe: medial surface, calcarine fissure (left)	Visual II	Occipital lobe (lateral): Superior occipital gyrus (right)	0.0007	AD > EMCI 0.67 (0.17) > 0.50 (0.20)
	Occipital lobe: inferior surface, Lingual gyrus (right)	Visual II	Occipital inferior gyrus (right)	0.0015	LMCI > EMCI; NC > EMCI 0.62 (0.12) > 0.49 (0.21); 0.62 (0.16)
Auditory	Temporal lobe: middle temporal gyrus (right)	Visual II	Occipital lobe (lateral): Superior occipital gyrus (left)	0.0025	AD < NC 0.11 (0.29) < 0.34 (0.20)
Basal ganglia	Caudate (right)	Dorsal left	Frontal lobe: Inferior frontal gyrus	0.0022	AD > NC 0.45 (0.19) > 0.25 (0.25)
	Subcortical gray nuclei: Caudate nucleus (left)	Dorsal left	Temporal lobe: middle temporal gyrus (left)	0.0022	AD > NC 0.39 (0.21) > 0.22 (0.23)
Thalamus	Sub cortical gray nuclei (right)	Ventrolateral prefrontal cortex	Frontal lobe: Inferior frontal gyrus triangular part (Left)	0.0008	AD > NC 0.50 (0.14) > 0.30 (0.20)

The table displays the ROI to ROI Functional connectivity (FC) results and the differences between groups. HC, healthy controls; EMCI, early mild cognitive impairment; LMCI, late mild cognitive impairment AD, Alzheimer’s disease; DMN, default mode network.

**Figure 3:**
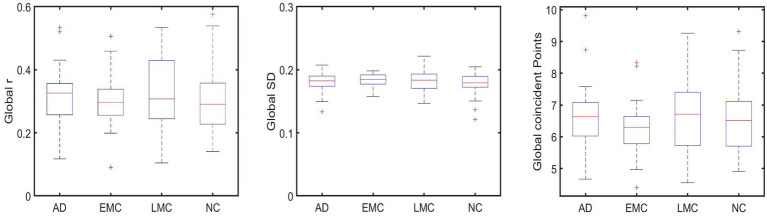
shows no significant differences between groups that were found globally in FC (subplot on the left), SWA (subplot in the middle), and PPA (subplot on the right).

To explore whether this pattern of connectivity was also present globally, the mean FC of all ROI-to-ROI correlations of each group was conducted. The results did not show a significant difference across groups (refer to Figure 3).

### Graph theory measures

Additionally, the clustering coefficient, degree, and global characteristic path length network measures were computed.

Patients with EMCI presented a significantly longer characteristic path length and a shorter mean degree in comparison to LMCI, AD, and HC. Results in the mean clustering coefficient did not show significant differences among groups (refer to Table 3).

**Table 3:** Graph theory measures.

Measure	HC $\bar{x}$ (SD)	EMCI $\bar{x}$ (SD)	LMCI $\bar{x}$ (SD)	AD $\bar{x}$ (SD)	Value of *p*	Group differences
Path length	1.568 (0.61)	1.606 (0.61)	1.501 (0.577)	1.516 (0.585)	0.05	EMCI > LMCI; EMCI > AD
Cluster coeff.	0.783 (0.11)	0.776 (0.11)	0.793 (0.04)	0.796 (0.11)	0.52	–
Degree	67.5 (23.98)	51.689 (23.71)	60.534 (24.11)	59.431(24.14)	0.02	EMCI<LMCI; EMCI<AD

It displays results in graph measures: the mean, standard deviation, and the significant differences between groups in path length, cluster coeff. (Clustering coefficient) and degree. The EMCI, early mild cognitive impairment group, presented a higher path length and a lower degree in comparison with the other groups.

### Sliding window correlation analysis

As expected, the variability when computing SD across windows using short windows, i.e., from 1 to 20 time points (3–60 s), was substantially higher, and the correlation was lower than when computing it for longer windows, i.e., 120–139 time points. Window lengths from 30 to 120 TR showed increased stability. Hence, the time series were divided into overlapping windows of 30 time points, i.e., 90 s to have as many windows as possible to ensure a good trade-off between sensitivity and robustness. Our windows are short enough not to miss FC fluctuations in our BOLD data and long enough to be robust and only real fluctuations. Previous studies also indicated enough variability and robustness in windows between 30 and 60 time points in rsfMRI studies (considering the frequency of the BOLD signal being 0.1–0.001 Hz; Esposito et al., 2019).

Greater differences in variability across groups were detected between several areas of the DMN and the attentional networks (executive and dorsal) between the left auditory cortex and the right dorsolateral frontal cortex, as well as between the right cerebellum right and the left sensory-motor cortex and between areas within the right cerebellum (refer to Table 4). Expectedly, the group that showed more variability in FC among windows is the EMCI group that showed a decrease in the static FC (analysis explained in the subsection above). Moreover, the AD showed a decrease in variability and presented an increase in sFC.

**Table 4:** Between group differences in mean ROI to ROI dispersion across windows using a one-way ANOVA.

Network	ROI	Network	ROI	P value	*Post hoc* $\bar{x}$ (SD)
Cerebellum	Cerebellum 7b (right)	Sensory motor	Postcentral gyrus (left)	0.0007	AD < EMCI 0.15 (0.07) < 0.21 (0.07)
	Cerebellum 8 (right)	Cerebellum	Cerebellum 7b (right)	0.0008	AD < EMCI; AD < LMCI 0.07 (0.07) < 0.12 (0.06); 0.07 (0.07) < 0.12 (0.07)
Basal ganglia	Caudate (right)	Dorsal right	Middle frontal gyrus orbital (right)	0.0016	HC < EMCI 0.17 (0.063) < 0.22 (0.045)
DMN	Superior frontal gyrus, medial orbital (left)	DMN	Angular gyrus (left)	0.0016	AD < EMCI 0.15 (0.05) < 0.17 (0.052)
	Superior frontal gyrus, medial orbital (left)	Dorsal left	Middle frontal gyrus, orbital (left)	0.0011	AD < EMCI 0.15 (0.06) < 0.17 (0.05)
Auditory	Temporal pole: superior temporal gyrus (left)	Dorsal right	Frontal lobe: Middle frontal gyrus (right)	0.0004	AD < EMCI 0.14 (0.052) < 0.19 (0.062)
	Rolandic operculum (left)	Sensory motor	Supplementary motor area (left)	0.0011	LMCI < EMCI; AD < EMCI 0.15 (0.04) < 0.21 (0.07); 0.16 (0.04) < 0.21 (0.07)
Executive function	Superior frontal gyrus orbital (left)	Dorsal left	Supramarginal gyrus (left)	0.0018	HC < EMCI 0.16 (0.05) < 0.22 (0.07)

Overlapping SWA, Sliding Window Analysis, results with a window size of 30 time points (TR = 3 s) and an increment of 1 time point for each slide. One-way ANOVA Significant differences between groups at a *p* < 0.05. HC, healthy controls; EMCI, early mild cognitive impairment; LMCI, late mild cognitive impairment AD, Alzheimer’s disease.

### Point process analysis

Some overlap in the results of the static FC and the PPA was detected (refer to Table 5). The LMCI and AD presented an increased FC between several brain regions in comparison to the HC and the EMCI. Specifically, the AD group, when compared with the LMCI, presented a higher FC between the cerebellum crus I (left) and the angular gyrus (left); an increased FC when compared with the EMCI, between the caudate (left) and the frontal gyrus (triangularis part); and also increased FC in comparison with HC between the insula (left) and the middle frontal gyrus (left), between the parahippocampus (right) and the insula (left), between the insula (left) and the inferior frontal triangular part (right), and between the thalamus (right) and the frontal inferior triangular part (left). The AD only presented a decrease in FC in contrast to LMCI between the cerebellum (right) and the amygdala (right).

**Table 5:** Differences in FC using the point process analysis.

Network	ROI	Network	ROI	Value of *p*	*Post hoc*
Cerebellum	Cerebellum 10 (right)	Basal ganglia	Amygdala (right)	0.0001	EMCI < LMCI; AD < LMCI 3.03 (2.14) < 6.3 (3.23); 3.61 (2.82) < 6.3 (3.23)
	Cerebellum Crus1 (left)	DMN	Angular gyrus (left)	0.0003	LMCI < AD 4.3 (2.71) < 7.05 (0.62)
	Cerebellum crus I (left)	Dorsal left	Middle temporal gyrus (left)	0.0015	EMCI < AD 7.68 (3.21) < 9.97 (3.52)
Visual III	Middle occipital gyrus (right)	DMN	Precuneus (right)	0.0012	LMCI < HC 7.8 (3.74) < 11.22 (3.53)
Basal ganglia	Caudate (right)	Dorsal left	Frontal lobe: Inferior frontal gyrus triangular part (left)	0.0006	EMCI < AD 5.34 (2.37) < 7.9 (2.93)
	Parahippocampal (right)	Auditory	Insula (left)	0.0014	HC < AD 3.71 (2.12) < 5.85 (2.74)
	Caudate nucleus (left)	Dorsal left	Inferior frontal gyrus, triangular part (left)	0.0018	HC < AD; EMCI < AD 5.51 (2.48) < 7.76 (3.40); 5.41 (2.47) < 7.76 (3.40)
Thalamus	Sub cortical gray nuclei: Thalamus (right)	Dorsal left	Inferior frontal gyrus triangular part (left)	0.0020	AD > HC 8.97 (3.08) > 6.31 (2.44)
DMN	Superior frontal gyrus, medial orbital (right)	Dorsal right	Parietal superior gyrus (right)	0.0012	HC < LMCI 7.14 (3.82) < 7.36 (3.69)
	Superior frontal gyrus medial orbital (right)	Dorsal right	Angular gyrus (right)	0.0016	EMCI < LMCI; HC < LMCI 6.51 (3.36) < 8.1 (3.35); 7.25 (3.70) < 8.1 (3.35)
Auditory	Insula (left)	Dorsal left	Inferior frontal gyrus triangular part (left)	0.0009	HC < AD 6.8 (2.72) < 9.58 (3.66)
	Insula (left)	Dorsal (right)	Inferior frontal gyrus triangular part (right)	0.0015	HC < AD 5.4 (2.88) < 7.14 (3.87)
Executive function	Superior frontal gyrus medial (left)	Dorsal (right)	Inferior frontal gyrus triangular part (right)	0.0014	EMCI < LMCI 6.96 (3.25) < 8.33 (3.21)

It displays the differences between groups in dynamic functional connectivity (dFC) between pairs of regions (ROIs) measured by points that surpass a threshold of BOLD signal activation of 1 standard deviation (SD). HC, healthy controls; EMCI, early mild cognitive impairment; LMCI, late mild cognitive impairment AD, Alzheimer’s disease; DMN, default mode network.

The LMCI presented a higher FC in comparison with EMCI between the cerebellum (right) and the basal ganglia and between the superior frontal gyrus medial (left) and the inferior frontal triangular part (right). The LMCI also presented a higher FC compared to HC between the parietal superior gyrus (right) and the orbital medial gyrus (right). The LMCI only presented a decrease in FC between the precuneus (right) and the middle occipital gyrus (right) in comparison to HC.

**Figure 4:**
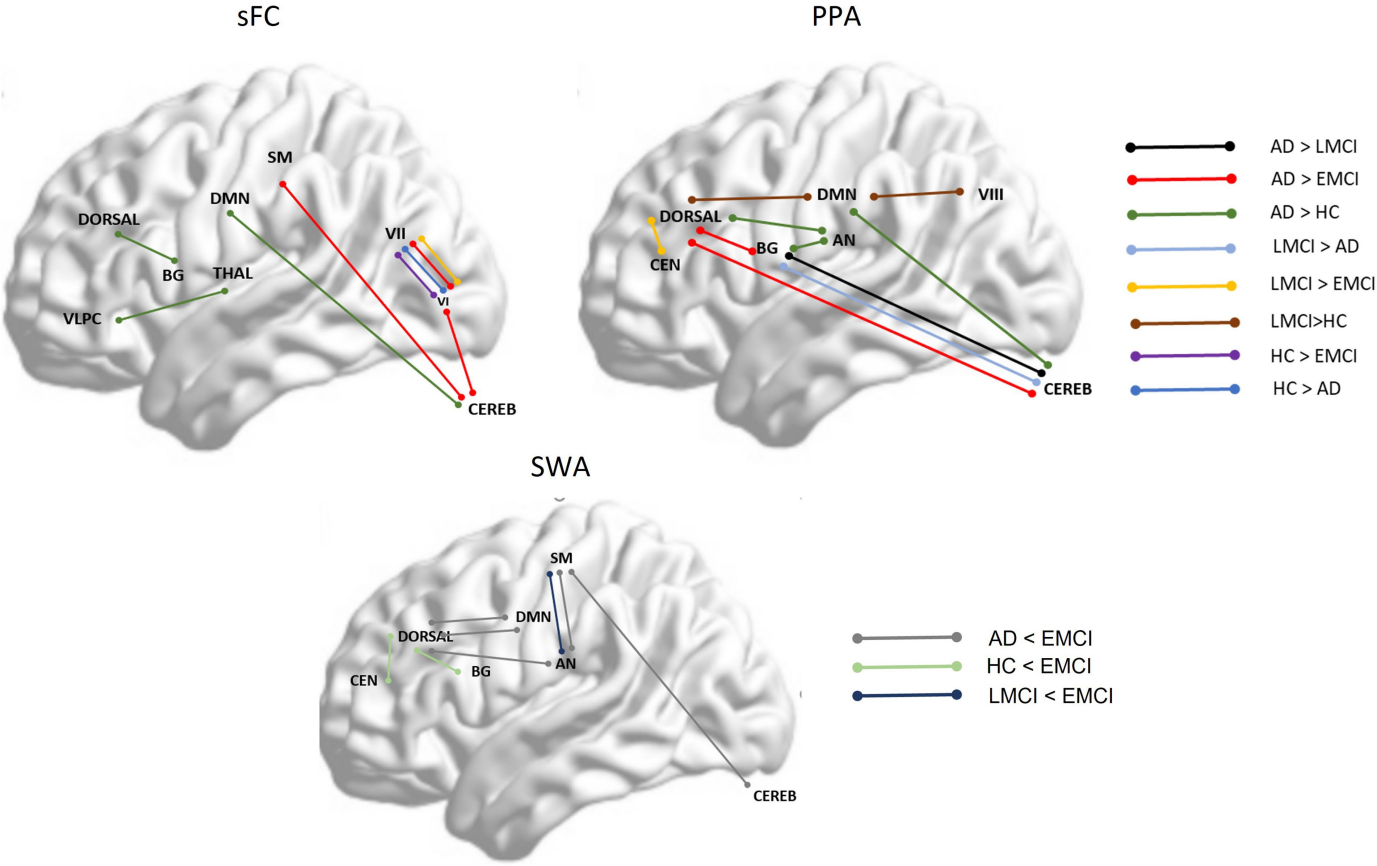
shows between-group significant differences between specific brain networks in static functional connectivity (sFC), point process analysis (PPA), and variability in functional correlation across windows applying sliding window correlation analysis (SWA). Dorsal, dorsal network; DMN, Default Mode Network; SM, Somatosensory network; VLPC, Ventrolateral prefrontal cortex; BG, Basal Ganglia; Thala, thalamus; VI, Visual network I; VII, Visual network II; Cereb, Cerebellum; CEN, Central Executive Network; AN, Auditory Network.

To summarize, Figure 4 displays a general view of the results obtained using FC, PPA, and SWA. As shown in the images, both the static FC and the PPA results showed an increase in connectivity between specific regions in the AD and the LMCI and a decrease in connectivity in EMCI. Additionally, the SWA results showed that variability in FC between brain regions across time is higher in the EMCI, which is statistically coherent with the fact that correlation or FC between regions is reduced in this group. The PPA was more sensitive in detecting changes across groups.

## Discussion

This study aimed to explore FC across stages of AD using the PPA and other classical methods. It was hypothesized that the connectivity in and between the DMN, SN, CEN, and VN would be altered in MCI and AD. Our results showed several differences across groups within and between the mentioned networks and other additional networks. Moreover, it was expected that there would be an increase in FC for EMCI but a decrease in AD for the mentioned networks. Unexpectedly, an increase in connectivity in LMCI and AD and a slight decrease in EMCI in contrast to the other groups were found. Finally, as expected, the PPA was the most sensitive method when capturing differences in FC across groups. A discussion of the results obtained using the different methods is provided below.

### Static FC and PPA: An increased FC in LMCI and AD

A reduction in mean functional connectivity between several brain regions, mostly in posterior and medial areas, as well as an increase in the FC variability across windows of time was expected in AD in comparison to EMCI and LMCI. However, our results showed a higher FC in LMCI and AD, but a decrease in EMCI in comparison to the other groups between several brain regions. This suggests a non-linear connectivity pattern that begins with optimal connectivity in HC, followed by a slight decrease in EMCI, and then by an enhancement of FC between several regions in LMCI and mild AD.

Some researchers used rsEEG (Bonanni et al., 2021) and rsfMRI and reported an increase in FC between multiple brain regions in prodromic AD and a decrease in AD. This mechanism is hypothesized by the authors to be caused due to GABAergic–glutamatergic dysregulation. Additionally, a postmortem study (Snowden et al., 2019) that analyzed brain tissue found that patients with AD presented a GABAergic increase and a glutamate decrease in AD in the inferior temporal gyrus, the most vulnerable region, to accumulate TAU tangles. Consequently, an inhibitory environment and a decrease in action potentials take place. Taken together, we would expect to observe an increase in connectivity in prodromic AD and MCI followed by a decreased FC in AD. In our study, however, a decreased FC was only observed in EMCI followed by an increased FC in LMCI and AD. The exception to this was for a pair of posterior regions that presented a decrease in connectivity in AD, i.e., the right middle temporal gyrus and right superior occipital gyrus. This increased FC in LMCI and AD was only seen in the current study by differentiating EMCI and LMCI, not often done in previous works. Moreover, in a postmortem study, patients with AD were older and presented moderate or severe AD, where decreased connectivity is more expected due to an increase in cellular death and atrophy. Furthermore, it is well-established that a high release of glutamate is neurotoxic and might be one of the causes of neuronal death (Maragos et al., 1987). Here, it can be hypothesized that the increased FC observed in LMCI and AD groups could be enhanced by a low GABAergic or inhibitory activity along with a high or excitatory activity in areas where TAU is not yet installed (e.g., thalamus, caudate, and/or cerebellum). On the other hand, early pathologic damage might explain the decreased FC in AD between the right middle temporal gyrus and the right superior occipital gyrus.

An increased FC has also been associated with mal(adaptive) rewiring of the brain after pathologic damage occurs. This idea is not mutually exclusive to the neurotransmitter dysregulation hypothesis, and the two mechanisms might co-occur in an additive fashion. Some authors have distinguished two types of hyperconnectivity rewiring after brain damage: local hyperconnectivity and global hyperconnectivity (Snowden et al., 2019). The former states that local alternative paths close to damaged brain areas are used to ensure that communication between networks is possible to preserve function. The latter refers to the brain using multimodal or rich nodes, independently of the localization of the brain damage, in order to ensure the maintenance of cognitive function. Following the popular stages of AD degradation as proposed by Braak and Braak’s (1995) research, the first areas damaged in AD (stages I and II) due to TAU deposition are the posterior ones, more specifically, the brain stem followed by the entorhinal. These areas are then followed by the limbic and whole neocortex in more advanced stages (III–IV), and then the hippocampus and anterodorsal thalamic nucleus. As the participants of our study present mild AD, this sign of increased FC in some posterior areas might indicate that certain regions of the posterior cortex could be affected but other areas are still preserved and can be recruited. A study showed increased correlations between occipital regions. Some investigators presented the same results regarding hyperactivity in AD in the middle occipital gyrus, lingual gyrus, and visual cortex and explained the findings as an adaptation of the brain networks in response to the disease (Wang et al., 2019).

Although several researchers have justified an increased FC in neurodegenerative diseases as an adaptive mechanism of neuroplasticity to protect behavior and cognition against brain damage, the mechanisms behind FC and its relationship with cognition and behavior are not well understood yet. Multiple factors play a role in FC, and at the time of writing, there is a lack of understanding of what is really causing a specific pattern of FC, i.e., what factors can explain an increased or decreased FC. Currently, interpretations of results in FC have been speculative. For instance, can an increase in FC in several areas be explained simply by structural disconnection and not by compensatory mechanisms? A study conducted by Patel et al. (2018) revealed that structural disconnection in patients with multiple sclerosis could explain an increase in rsfMRI (Wang et al., 2019). Whether this increase in FC is due to neural compensatory mechanisms or not needs to be further investigated using multimodal neuroimaging techniques.

### Additional measures performed: Graph theory measures

Patients with EMCI showed a longer characteristic path length in contrast to the patients with LMCI and AD, probably reflecting a need for EMCI to connect inter-regionally due to local damage. In comparison, in LMCI and AD, there might be more widespread pathology and more areas connected in a more disorganized way and presenting shorter paths. A shorter characteristic path length in HC is probably a sign of efficiency in connectivity between long distant areas, while in AD, shorter paths might mean disrupted connectivity, and the whole brain is more connected but in a disorganized manner, i.e., a decrease in metastability. A study reported disrupted global metastability in AD (Córdova-Palomera et al., 2017). Other studies reported longer mean characteristic path lengths in MCI (Wang et al., 2013).

It can be speculated that in LMCI and at the beginning of AD, the brain might be rewiring in a widespread manner, finding local and global alternative paths to maintain cognitive functioning. This adaptive and short-term solution might incur, however, a high cost for the network, as usually, any alternative paths that involve rich hubs generate a proper environment to facilitate the formation and accumulation of TAU protein tangles (Bonanni et al., 2021).

### Sliding windows correlation analysis (variability among windows)

It has been reported that patients with pathology might present more variability in the BOLD signal over time. The SD of the correlation metrics across windows allowed us to see the variability in FC between pairs of regions. Usually, regions with greater variability imply a lower correlation (Rolls et al., 2021).

Significant differences in variability across windows were found between groups. Results showed that the EMCI presented a greater variability over time in most connections. This finding is aligned with the static FC results that showed a decreased correlation between several brain regions. Most of the ROIs detected are similar for static FC and PPA analysis.

### Specific brain regions affected in AD and LMCI with decreased FC (static FC analysis, SWA, and PPA)

Besides this decrease–increase in FC patterns, the specific brain regions that presented a sharp abnormal connectivity show consistency with other studies. For instance, several studies have reported a decrease in FC in EMCI between the thalamus (left/right) and frontal gyrus and between the temporal and occipital gyrus (Cai et al., 2015). Additionally, a study showed that AD participants presented a larger caudate nucleus volume in AD, probably reflecting a compensation mechanism of damage in the closest areas such as the hippocampus. Pathology in the hippocampus could explain an increased stimulation of the caudate nucleus, causing an increase in volume and FC between it and the cortical brain regions (Persson et al., 2018).

On the other hand, the cerebellum was classically thought to be unaffected in MCI and AD (Chételat et al., 2008). However, gray matter atrophy in the cerebellum and pathological changes, such as beta-amyloid deposits and neurofibrillary tangles, have been observed (Ciavardelli et al., 2010; Córdova-Palomera et al., 2017). The current results showed abnormal connectivity in the cerebellum and between the cerebellum and other regions (cerebellum crus II and DMN, sensory-motor and cerebellum VII, and cerebellum 8 and cerebellum 7b). Other studies also found aberrant FC within the cerebellum (Cai et al., 2015) and between the Crus II and the DMN (Toussaint et al., 2014).

The precuneus, a specific region of the DMN, is a relevant hub that enhances connectivity between several brain regions. A decreased FC between this and other regions has been associated with changes in the brain’s vulnerability in early Alzheimer’s disease. Our PPA results showed a decreased FC in LMCI in contrast with HC (Nelson et al., 2009; Yokoi et al., 2018). A decreased FC of the precuneus might be the cause of increased FC in regions next to this region (local hyperconnectivity hypothesis) and far from this region (global hyperconnectivity hypothesis).

### The added value of SWA and the PPA to the static FC

Although our results showed some overlap or complementarity across methods, for instance, areas or networks, there were also differences across methods. It is worth noting that the PPA was able to detect more changes across groups.

Some areas that showed aberrant FC detected by means of the SWA and the PPA were not detected in the static FC. For instance, the AD presented less variability and more synchronicity between the auditory and middle frontal gyrus in comparison to the HC and the EMCI. In addition, the AD group, in contrast to the HC and the EMCI, presented increased FC and decreased variability between regions of the auditory network, e.g., insula, superior temporal, and the dorsal (right), such as the inferior frontal triangular part and middle frontal gyrus. Finally, the EMCI presented a decreased connectivity in contrast to the LMCI between the executive function network and the dorsal.

When comparing the SWA and the PPA, a higher variability from the SWA was obtained in the EMCI in comparison to the HC between a region in the executive function network and the dorsal, and in the PPA, a higher correlation in the LMCI than in the EMCI. These couple of findings made us hypothesize that there is stronger synchronicity between these areas in LMCI and HC but less in EMCI. Without performing the PPA, we could not have seen the difference in FC between these regions.

Finally, the PPA was the method that allowed us to have a more comprehensive or complete view of the aberrant connectivity across the different stages of the disease. Besides, we could gauge the subtle changes across groups like an increase in LMCI in comparison to EMCI between the dorsal network and the CEN, as well as an increase and decrease in FC between some ROIS of the cerebellum and the basal ganglia in LMCI and AD.

This study presents some limitations. First, the optimization of window parameters to conduct the SWA as well as the threshold to binarize the FC matrix and extract graph measures were carefully selected after a literature revision and a variability and correlation assessment for each window size; nonetheless, the optimization of parameters in FC research is an issue that should be further investigated with novel analysis. Second, the structural parcellation method used in the present study, the AAL, is commonly used for task and rsfMRI studies. Although some studies have used connectivity-driven parcellations and have shown more consistency with the subjacent resting state connectivity, anatomical parcellations yield the same or even better delimitation of cortical areas, which is relevant for network analysis. Given these claims, the selection of a specific parcellation over others will not impact many of the results. As suggested by Arslan et al. (2018) in their systematic comparison of parcellation methods, researchers performing network analysis should use any parcellation method available. Third, participants with eMCI, lMCI, or AD presented a clinical diagnosis and their memory was objectively affected. However, a biological characterization of the participants was not conducted and would enable the generalization of findings.

Comparing results across methods was a difficult task because some results were similar across methods, but several differences could also be found. This happened because the methods used measured different FC constructs: static FC, variability in FC, and dFC. Hence, expecting similar global results in terms of differences between some networks or patterns of increased or decreased connectivity in several brain regions at a specific stage of the disease was logical; however, expecting similar findings across methods, at a finer scale, i.e., small regions was not achievable.

## Conclusion

Our results showed that the EMCI presented a similar but slightly decreased FC to HC in several brain areas, while the LMCI and mild AD presented an increased FC in several regions. These results suggest that the pathology is less dispersed in EMCI and the brain configuration is similar to the HC. When the pathology advances in LMCI and AD, the brain might react by compensating, using the available resources such as the recruitment of alternative paths. This might enhance high glutamate and low gabaergic activity next to regions where pathologic proteins are installed and in rich hubs such as the thalamus. The analysis performed provided some overlap in the results. For instance, they showed an increased FC in LMCI and AD involving a specific frontal region, i.e., the frontal inferior triangular (left) and posterior regions mostly within the visual network, the DMN, the auditory, cerebellum, basal ganglia, and thalamus. However, as expected, the results also show differences in the areas affected when applying different methods. This was expected as the literature review shows this heterogeneity because what we are measuring across methods fits under the same “FC” umbrella, but it is not the same, i.e., static FC, variability in FC, and dFC are different measures. Finally, SWA and PPA added new results, and this last method is the most efficient when dealing with datasets and sensitive differentiating changes across the stages of the disease.

Future studies should include a larger sample and diverse AD groups that reflect all the stages of the disease, i.e., amnestic EMCI, amnestic LMCI, mild AD, moderate AD, and severe AD. Additionally, longitudinal studies that track the connectivity across years, as well as postmortem studies to explore the brain tissue of different patients that died at different stages of the disease would be highly useful to understand the relationship between neurotransmitters, pathological proteins, and FC at the different stages of AD. Finally, novel machine-learning approaches could be used to compare and integrate the findings.

## Data availability statement

Publicly available datasets were analyzed in this study. This data can be found at: adni.loni.usc.edu.

## Author contributions

LP conducted the data preprocessing, analysis and wrote the first draft of the manuscript. IC and AS contributed to the design of the study. IC provided technical support in the data preprocessing and analysis. IC, AS, and P-OS contributed to manuscript revision, read, and approved the submitted version. All authors contributed to the article and approved the submitted version.

## Funding

Financial support was provided by the MICINN (Spain) grant PID2021-125534OB-I00 PID2021-125534OB-I00. Data used for this study were funded by the Alzheimer’s Disease Neuroimaging Initiative (ADNI) (National Institutes of Health Grant U01 AG024904) and DOD ADNI (Department of Defense award number W81XWH-12-2-0012). ADNI is funded by the National Institute on Aging, the National Institute of Biomedical Imaging and Bioengineering, and through generous contributions from the following: AbbVie, Alzheimer’s Association; Alzheimer’s Drug Discovery Foundation; Araclon Biotech; BioClinica, Inc.; Biogen; Bristol-Myers Squibb Company; CereSpir, Inc.; Cogstate; Eisai Inc.; Elan Pharmaceuticals, Inc.; Eli Lilly and Company; EuroImmun; F. Hoffmann-La Roche Ltd. and its affiliated company Genentech, Inc.; Fujirebio; GE Healthcare; IXICO Ltd.; Janssen Alzheimer Immunotherapy Research & Development, LLC.; Johnson & Johnson Pharmaceutical Research & Development LLC.; Lumosity; Lundbeck; Merck & Co., Inc.; Meso Scale Diagnostics, LLC.; NeuroRx Research; Neurotrack Technologies; Novartis Pharmaceuticals Corporation; Pfizer Inc.; Piramal Imaging; Servier; Takeda Pharmaceutical Company; and Transition Therapeutics. The Canadian Institutes of Health Research is providing funds to support ADNI clinical sites in Canada. Private sector contributions are facilitated by the Foundation for the National Institutes of Health (www.fnih.org). The grantee organization is the Northern California Institute for Research and Education, and the study is coordinated by the Alzheimer’s Therapeutic Research Institute at the University of Southern California. ADNI data are disseminated by the Laboratory for Neuro Imaging at the University of Southern California.

## Conflict of interest

The authors declare that the research was conducted in the absence of any commercial or financial relationships that could be construed as a potential conflict of interest.

## Publisher’s note

All claims expressed in this article are solely those of the authors and do not necessarily represent those of their affiliated organizations, or those of the publisher, the editors and the reviewers. Any product that may be evaluated in this article, or claim that may be made by its manufacturer, is not guaranteed or endorsed by the publisher.

## Footnotes

^1^ http://adni.loni.usc.edu/data-samples/access-data/
