# Supplementary material for: Increased functional connectivity patterns in mild Alzheimer’s disease: A rsfMRI study
Source: Front Aging Neurosci. 2023 Jan 9;14:1037347. doi: 10.3389/fnagi.2022.1037347 (PMC9869068; doi:10.3389/fnagi.2022.1037347)
Supplement: Supplementary file 1 [file Table_1.DOCX]

Supplementary Material

## Supplementary 1. Optimization of thresholding based on mean degree and small-world estimation

After computing the FC, the correlation (FC) matrices were transformed into binary matrices to compute graph measures (raw correlation values were used). Until the moment it does not exist an accurate way to choose a threshold to study brain networks, however, several researchers using rsfMRI datasets used a threshold between 0.2 to 0.5 as it provided scale freeness and enabled differentiation of groups. For this reason, an analysis of our data to ensure these two characteristics was conducted. In this analysis, we assessed the global degree using different correlation thresholds, i.e., from 0.1 to 0.5 at intervals of 0.01. A threshold of 0.3 was finally chosen because the mean degree when using this threshold is not too high not too low, satisfying the smallworldness feature of the brain, this way those nodes that are highly connected with several regions and the ones that are only connected to a few regions were captured. Moreover, this threshold allows a good discrimination across groups (see plot below). Those ROI-to-ROI connections that exceeded a threshold of 0.3 (Pearson’s correlation coefficient raw value) were set to 1, whilst those below 0.3 were set to 0.

**
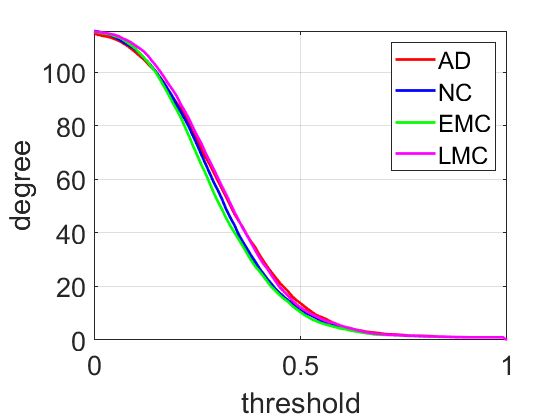
**

**Note.** *The figure shows the mean degree (mean number of nodes connected to another node), y axis and the threshold set, x axis.* *Smallworldness is most preserved at a threshold between 0.3 and 0.5. Showing that the mean degree is not too high, not too low, revealing a few highly connected networks and many with a low degree. It also shows that differentiation between groups is more evident when binarizing the matrix using one of this range of thresholds.*

Researchers in the following studies used a similar threshold to binarize FC matrices before performing graph theory:

- Ahmadi, H., Fatemizadeh, E., & Motie-Nasrabadi, A. (2020). Multiclass classification of patients during different stages of Alzheimer’s disease using fMRI time-series. *Biomedical Physics and Engineering Express*, <https://doi.org/10.1088/2057->
- Aurich, N. K., Filho, J. O. A., da Silva, A. M. M., &#38; Franco, A. R. (2015). Evaluating the reliability of different preprocessing steps to estimate graph theoretical measures in resting state fMRI data. *Frontiers in Neuroscience*, (FEB), <https://doi.org/10.3389/fnins.2015.00048>
- Ng, A. S. L., Wang, J., Ng, K. K., Chong, J. S. X., Qian, X., Lim, J. K. W., Tan, Y. J., Yong, A. C. W., Chander, R. J., Hameed, S., Ting, S. K. S., Kandiah, N., & Zhou, J. H. (2021). Distinct network topology in Alzheimer’s disease and behavioral variant frontotemporal dementia. *Alzheimer’s Research and Therapy*, *13*(1). https://doi.org/10.1186/s13195-020-00752-w
- Ye, M., Yang, T., Qing, P., Lei, X., Qiu, J., & Liu, G. (2015). Changes of functional brain networks in major depressive disorder: A graph theoretical analysis of resting-state fMRI. *PLoS ONE*, *10*(9). <https://doi.org/10.1371/journal.pone.0133775>

## Supplementary 2. Steps Point Process Analysis

Below, an explanation of the three steps conducted in the PPA are explained:

1. **Signals thresholding** (1*sd*). First, signals were thresholded, as shown in the figure below.


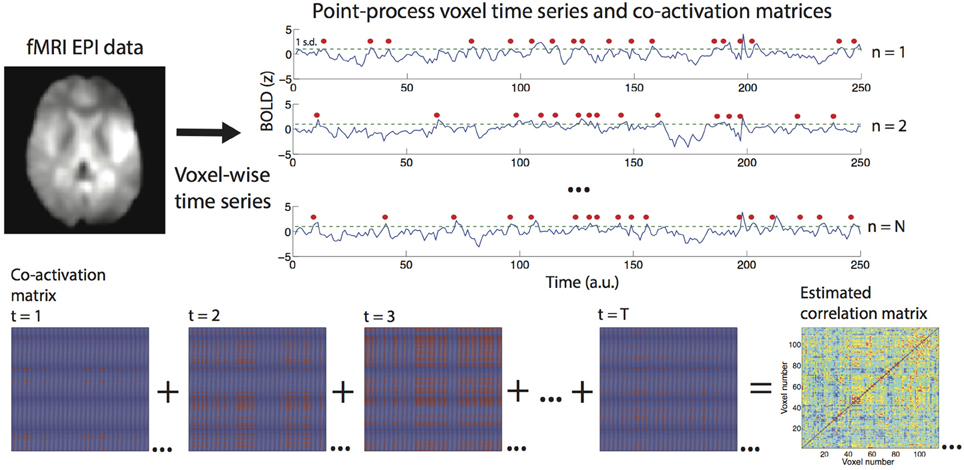

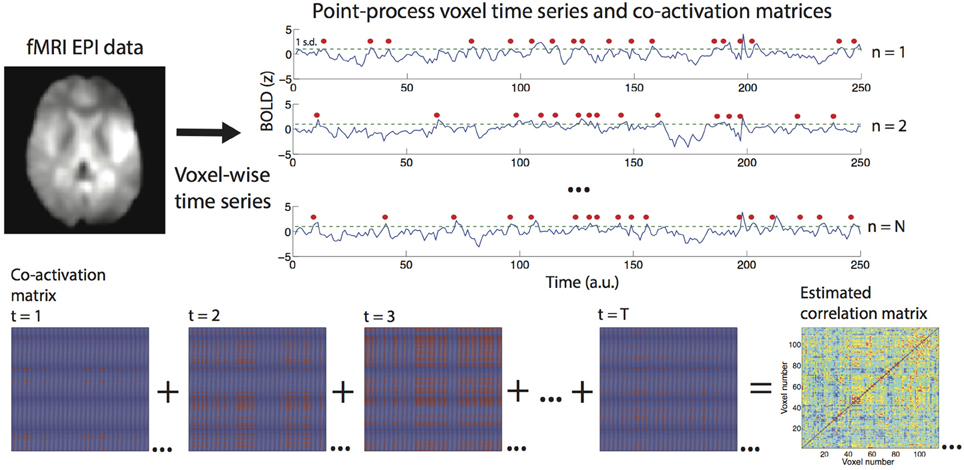


Figure extracted from Cifre et al., 2017 (reference below).

1. **Co-activation matrices:** then, all those points of the timeseries that surpassed 1 *SD* of the BOLD signal were given a 1 (relevant) and the rest were given a 0 (non-relevant), then co-activation matrices for each timepoint were generated, e.g., in timepoint 1 of ROI 1 and ROI 2 the signal is relevant as both surpass 1 *SD*, in timepoint 1 ROI 1 and timepoint 1 ROI 3 in time the signal is not relevant as it does not surpass the threshold (< 1 *SD*…). This is conducted for all the 140 timepoints for each 116 pairs or regions. The figure below displays whether each pair of ROIs are coincident in each timepoint.


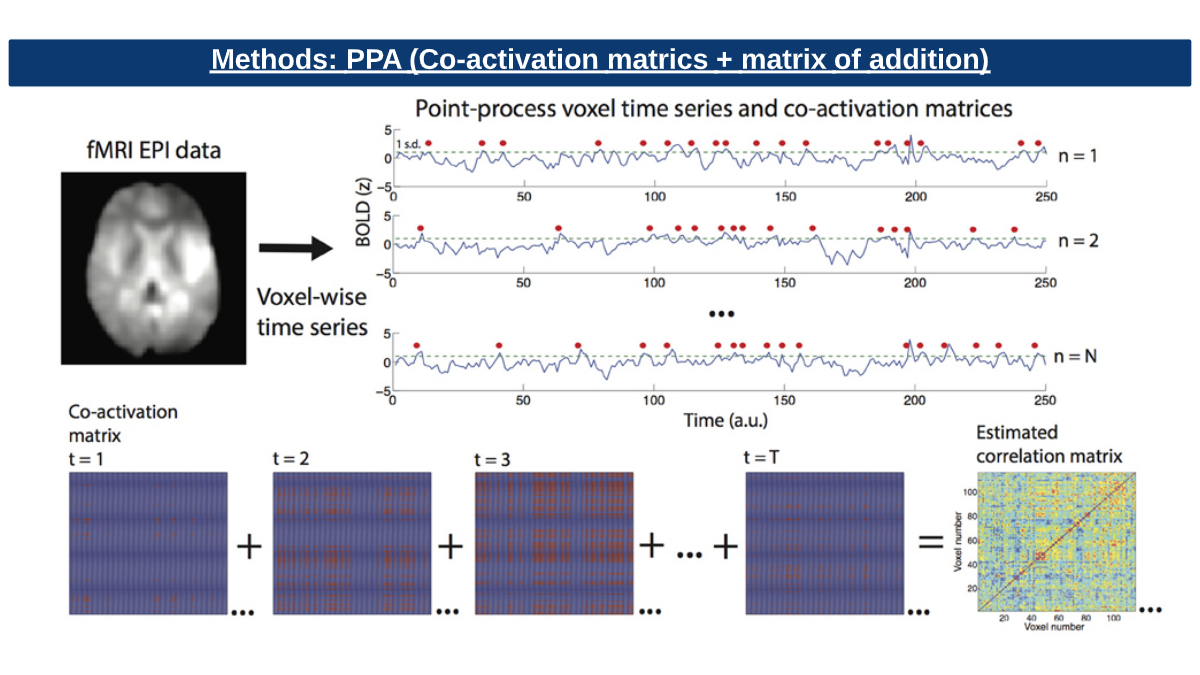


Figure extracted from Tagliazucchi et al., 2016 (reference below).

1. **Matrix of addition:** Then, a matrix of addition was extracted for each pair of ROIs, i.e., connectivity between ROI 1 and ROI 2 in PPA is acquired by adding all the timepoints that were relevant in all the time series. Similar to a correlation matrix, but here instead of correlation between pairs of regions we are computing a summation of the number of relevant points thorough the timeseries.


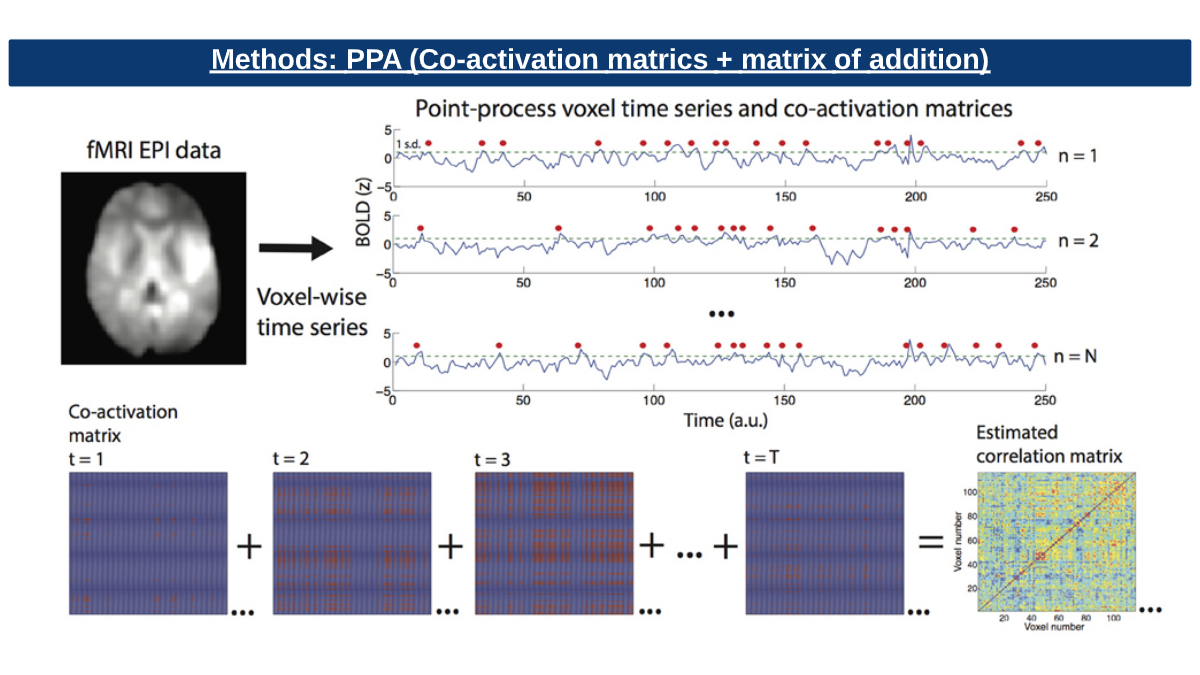


Figure extracted from Tagliazucchi et al., 2016 (reference below).

See these references below for a comprehensive explanation of the method:

Cifre, I., Zarepour, M., Horovitz, G., Cannas, S. and Chialvo, D. (2017). On why a few points suffice to describe spatiotemporal large-scale brain dynamics, arXIv. https://doi.org/10.48550/arxiv.1707.00759.

Cifre, I., Zarepour, M., Horowitz, S.G., Cannas, S.A. and Chialvo, D.R. (2020). Further results on why a point process is effective for estimating correlation between brain regions, *Papers in Physics*, 12(June), pp. 1–8. <https://doi.org/10.4279/pip.120003>.

Tagliazucchi, E., Balenzuela, P., Fraiman, D. and Chialvo, D.R. (2012). Criticality in large-scale brain fmri dynamics unveiled by a novel point process analysis, *Frontiers in Physiology*, 3 FEB. <https://doi.org/10.3389/fphys.2012.00015>.

Tagliazucchi, E., Siniatchkin, M., Laufs, H., & Chialvo, D. R. (2016). The voxel-wise functional connectome can be efficiently derived from co-activations in a sparse spatio-temporal point-process. *Frontiers in Neuroscience*, *10*(AUG). https://doi.org/10.3389/fnins.2016.00381.
